# Exploring challenges and perceptions in the learning environment: an online qualitative study of medical students

**DOI:** 10.1186/s12909-024-05116-8

**Published:** 2024-02-14

**Authors:** Mohammed Almansour, Fatmah Almoayad

**Affiliations:** 1 Department of Medical Education, College of Medicine, King Saud University, Riyadh, 11461 Saudi Arabia; 2 Department of Health Sciences, College of Health and Rehabilitation Sciences, Princess Nourah Bint Abdulrahman University, P.O. Box 84428, Riyadh, 11671 Saudi Arabia

**Keywords:** Learning environment, Medical school, Medical student, Medical education, Student experience, Saudi Arabia, Qualitative research, Thematic analysis, Faculty role

## Abstract

**Background:**

The teaching and learning environment (TLE) in medical schools is critical for shaping the outcomes and competencies of graduates. Research on TLE has highlighted its influence on student learning approaches and outcomes, yet gaps remain, particularly in qualitative insights, especially in Saudi Arabian contexts. This study aims to explore the students’ experiences and perceptions of the TLE in a new medical college.

**Methods:**

This qualitative study consisted of a total of five focus group discussions (3consequtive sessions for male group and two for female group) conducted virtually using the Zoom videoconferencing application. All the discussion sessions took place during a lockdown because of the COVID-19 pandemic between December 2020 and February 2021. Each session lasted for 45–60 min. Each group was formed of 4–5 students from different academic levels in the Majmaah medical school, which was established 10 years ago and is located in a small city. After “*verbatim transcription”* of the sessions was made, a framework thematic analysis of the data was performed using the NVivo software.

**Results:**

The study results revealed that various elements, such as educational content, faculty roles, and personal factors, collectively influenced the students’ educational experiences. The students valued educational relevance and autonomous decision-making. The multifunctional role of faculty as mentors, evaluators, and resource providers was considered essential in enhancing academic experiences. Additionally, there was an evident need to improve the physical learning environment and facilities to adapt to emerging educational needs. These results align with existing literature, emphasizing the integration of theory and practice and the significant impact of faculty roles in academic experiences.

**Conclusion:**

The findings suggest that medical colleges should involve students more in decision-making related to their education and ensure the practical relevance of the educational content. Establishing open communication channels between students and faculty who act as mentors and evaluators is also essential. Furthermore, enhancing supportive infrastructures, such as mental health and financial services, and promoting extracurricular activities are crucial for fostering a more effective and nurturing learning environment.

**Supplementary Information:**

The online version contains supplementary material available at 10.1186/s12909-024-05116-8.

## Introduction

Teaching and learning environment (TLE) in medical schools as attracted growing attention in the academic community, reflecting its central role in shaping the educational outcomes and professional competency of graduates [1]. Understanding the TLE in medical schools involves exploring several factors, such as administrative arrangements, curriculum design, assessment methods, student–teacher interaction, resources, facilities, and psychosocial elements [2–4].

Since the late 1970s, the study of the impact of the TLE on medical students has grown. Initially focusing on deep and surface learning approaches, the research has expanded. It now encompasses diverse learning strategies and styles, highlighting the influence of the TLE on student outcomes [5]. Ramsden and Entwistle [6] laid the groundwork for studying the relationship between approaches to learning which refers to the various ways in which students engage with their educational experiences and perceptions of the learning environment students’ views and opinions about the various aspects of their educational setting. In their study, they identified three main factors in approaches to studying: orientations toward personal meaning, reproducing, and achieving.

Despite advancements in TLE research, some contradictory evidence exists regarding the relationship between TLE characteristics and students’ learning approaches [7]. Additionally, there are indications of cultural differences in student learning approaches [8], which further the understanding of these dynamics.

Faculty play a crucial role in cultivating a positive TLE by encouraging peer collaboration, stimulating discussions, and fostering positive perceptions [9]. These aspects are vital for both learning and academic well-being, as indicated by major educational bodies [10–12].

A supportive TLE has been associated with the overall success of an educational program and the development of competent graduates, influencing aspects such as feelings, mood, and the prevention of burnout [13–16]. Several studies have demonstrated the significance of monitoring students’ perceptions as an effective method for evaluating and improving the TLE [12, 17–19]. For example, learning communities in medical schools have been linked with positive perceptions of the learning environment [20], while factors such as quality teaching, faculty support, and self-directed learning spaces are influential in shaping these perceptions [19–21]. The ongoing evaluation and adaptation of TLEs in collaboration with teachers and students is crucial for improving medical education.

Student surveys have been widely used by institutions across the world as an evaluation tool to assess teaching quality and learning experiences [22]. In Saudi Arabia, multiple studies have employed the Dundee Ready Education Environment Measure (DREEM) to understand students’ perceptions of their educational environment. Recent regional studies have provided new insights into undergraduate medical students’ learning styles and perceptions [23, 24]. However, these studies have also revealed varied perceptions in Saudi institutions. Some have reported positive changes after moving to new campuses. Others expressed concerns about teaching methods, student–teacher relations, and stress [25–27].

While global research offers a rich array of qualitative studies that intricately explore the TLE [7, 28, 29], there is a noticeable gap in the local literature, with Saudi Arabian studies predominantly relying on quantitative data. An emphasis on quantitative methodologies provides a broad overview but often lacks the in-depth insights that qualitative methods can bring, particularly in understanding the lived experiences of students [30].

Research conducted at the College of Medicine at Majmaah University has shed light on generally favorable perceptions of students toward the TLE. The study by Almansour, AlMehmadi [31] identified challenges in acquiring knowledge and developing subject-specific skills, particularly during the basic science years. Specific areas identified as needing improvement included organized studying, communicating knowledge, and effort management. Notably, differences in satisfaction levels between clinical and basic science students, as well as gender-specific perceptions, noted in the research.

Building on the insights provided by the prior mentioned study by Almansour AlMehmadi [31], the current study aimed to comprehensively explore the experience of students about teaching and learning environment at the College of Medicine at Majmaah University. By exploring into their lived experiences and perceptions, this research seeks to fill the gap in the local literature by providing qualitative data on the TLE. The insights gained will contribute to tailoring the learning environment more effectively to meet the diverse needs of the student population. This will ultimately contribute to the creation of an optimally supportive educational environment.

## Methods

### Study design and participants

This observational study involved medical students at the College of Medicine, Majmaah University, Al-Majmaah, Riyadh Region. A focus group discussion method was employed to deeply explore the impact of the teaching and learning environment on the student’s learning experiences.

### Setting and curricular framework

Majmaah University is an evolving educational institution in the region. It was established in 2009. Its college of medicine has adopted an outcome-based hybrid curriculum. Students must complete a six-year medical school program before they enroll in a one-year mandatory internship to obtain their bachelor of medicine/bachelor of surgery (MBBS) degree. The first year (phase 1) in medical school is preparatory, during which the students study chemistry, physics, and the English language. The following two and a half years start (phase 2) with system-oriented basic medical science modules and are followed by two and a half years (phase 3) of clinical clerkship in which students rotate between a major and sub-specialties with a main focus on practical clinical sessions. During all phases, evaluation includes theoretical and practical exams held after each module. This is followed by a mandatory internship year (phase 4) comprised of clinical rotations in medicine, surgery, obstetrics and gynecology, pediatrics, and emergency medicine. in addition to two elective clinical rotations. The interns are evaluated by their direct supervisors at the end of each rotation on a pass/fail basis, and they are eligible for the Saudi Medical Licensing Exam (SMLE), which is held twice a year.

### Sampling and recruitment

The participants were recruited for two focus group discussions during the last three months of the 2020/2021 academic year. The total combined number of male and female students in the college at the time of the study was 324. Each group comprised 4–5 participants who had previously participated in quantitative research conducted earlier in their college experience by the same researcher and his team. Both groups consisted of heterogenous type of participants from all academic levels as shown in Table 1. Following the local cultural norms in Saudi Arabia, the male and female focus groups were kept separate during their participation. A student affairs employee was asked by the researcher to recruit five students from the previous participants to join the two focus group discussions.

### Data collection

Data were collected between December 2020 and February 2021. General demographic information sheets were completed by the participants upon recruitment. The focus group discussions were led by the principal investigator. The researcher conducted the focus group discussions virtually through the Zoom videoconferencing application. The recording of all sessions was done through ZOOM videoconferencing features of recording. All focus group discussion sessions took place during a lockdown because of the COVID-19 pandemic, which mandated the use of technology to conduct the sessions. The main researcher, an observer from the research team (two sessions only), and the participants were the only individuals in the Zoom meeting. The discussions were audio-visually recorded and lasted between 45 and 60 min.

The participants were given the choice of speaking Arabic (the native language of all the participants) or English. The discussions were led by a topic guide that was developed based on the aims of the study, which was a deep exploration of our previous questionnaire-based study with the same population (See Additional file 1.) Flexibility was observed by following the structured topic guide to allow the emergence of new themes and in consideration of the interactive nature of the focus group.

The discussion started with a general question, “What is your experience of the teaching and learning as a medical student in the College of Medicine at Majmaah University?” This was followed by a discussion involving some of the learning environment issues identified in the previous study.

### Data analysis

The focus group recordings were transcribed professionally by external individuals. To preserve the meaning, the transcripts were analyzed in their original language, and only quotes (as presented in this article) were translated into English. Two individuals (the main researcher and an independent research assistant) were involved in coding the data. The data analysis process was based on the thematic analysis approach [32], which involves a six-step process: (1) familiarizing yourself with the data, (2) generating initial codes, (3) searching for themes, (4) reviewing themes, (5) defining and naming themes, and (6) producing the report. Themes were developed based on the identified patterns of the codes. Coding was completed using emergent themes, which were investigated until saturation was achieved. The themes were derived from the collected data and the topic guide, which was partially developed from the previous study. The NVivo software version 11.4.2 was used for data analysis. Using the software increases the efficiency of data organization and retrieval. Familiarization, descriptive coding, basic analysis, and interpretation are the steps followed in data analysis and quotes from the discussions were used to support the themes.

## Results

Five focus group discussions were conducted during the course of the study. Three discussion sessions involved the male student group (same participants complete the three sessions), and two sessions were held with the female student group (same participants complete the two sessions). Five male students and four female students voluntarily participated in each group, and they all continued to the conclusion of the study. The participant demographic data are shown in Table 1. This shows that they were well distributed in regard to educational level. Both the preclinical and clinical phases of the MBBS program were well represented.

**Table 1:** Demographic data of the focus group participants

Participant	Year	Gender	Age
P1M	4th	Male	23
P2M	5th	Male	24
P3M	2nd	Male	22
P4M	3rd	Male	22
P5M	Intern	Male	27
P6F	1st	Female	19
P7F	4th	Female	22
P8F	3rd	Female	22
P9F	5th	Female	23

The discussion sessions raised many issues about the learning environment in the local context of the college of medicine. The approach the groups used in handling the discussions was generally analytical problem-solving, and in some situations, it was more oriented toward generating recommendations, such as practical steps, to improve the learning environment. Three major themes emerged from the discussion sessions: (1) educational factors, (2) faculty-related factors, and (3) social and personal factors. Each theme contained a number of sub-themes (as shown in Table 2).

**Table 2:** Qualitative data analysis categories

Theme	Categories	Codes
1. Educational factors	1.1. Decisions about the importance and relevance of their learning	Gap between theory and practice Teaching strategies Topics
	1.2. Relevance of the curriculum structure to students’ learning	Modules Scheduling Timing
	1.3. Role of self-reflection	Reflecting on learning Exposure Thinking Feeling Relating
	1.4. Extracurricular activities	Clubs Activities Forums
	1.5. Academic atmosphere and resources	Buildings Atmosphere Outdoors Library Clinical training
2. Faculty-related factors	2.1. Role as a resource person	Knowledgeable Cooperative Clinical skills
	2.2. Role as an evaluator (feedback provider)	Feedback Assess Weaknesses Strengths
	2.3. Role as mentor	Mentor Meetings Guide
3. Social and personal factors	3.1. Educational challenges	Problems Time constrains Struggle Pressure
	3.2. Students’ interactions	Students’ interactions Assistance Groups Relationships Fights
	3.3. Psychological and financial support	Psychological consultations Clinic Discounts Shops

### Educational factors

#### Decisions about the importance and relevance of their learning

The students valued teaching methods that bridged the gap between theory and practice, which made their learning more relevant to real situations and practice. For example, the participants made the following comments:

*‘Roleplaying is simulating the things that we studied theoretically, and this information won’t stick in our heads until we see it in reality… such as rare conditions, it is almost impossible, and we may never see some of them during practice, and that can be beneficial. Generally, I love simulating, and personally, I always make sure I participate in them.’* [P1M].

*‘The Case Discussions (CDs), which in general have helped me and made me think, especially in differential diagnosis. I wish it was in all the years, not just from the fifth year.’* [P1F].

They also emphasized their interest in deciding about their learning, such as which important topics are needed by the students to ensure their autonomy in the learning environment. This point was in the context of being able to make recommendations rather than changes in actual practice. For example, one participant (P4M) stated,

Not just the doctor can decide roleplay topics, the students themselves can choose as well.

#### Relevance of the curriculum structure to students’ learning

The feeling of being stressed by the compact courses and the required learning material in the curriculum was shared by many students. Although some students expressed views similar to P3F:

I really like the college’s plan…. It is an excellent point, and instead of studying the diseases all at once and getting me lost, it has been divided into two sections. Even though in the sixth year, there is huge pressure on me.

Some students thought that the distribution of courses, materials, and skills had a rationale behind it. However, some courses seemed less relevant to other students, as shown in the following examples:

*‘In one module, I felt that it was not that important’*. [P4F]

*‘Last year was the worst with the pressure of the modules and some doctors… sometimes, they don’t accept that we take modules for two weeks, and I have midterms every week. We face huge psychological pressure’* [P3F].

#### Role of self-reflection

Reflections on learning were expressed by some students. They often related it to their phase or level of clinical exposure. For example, the following comments were made:

*‘I think I touched on this in the clinical phase more than basic science phase. I reflected on my learning about 30% in the basic phase and 70% in the clinical phase… when I knew things and I could relate to them, and I had the ability to answer questions from family and friends.’* [P4M].

*‘It depends on our exposure. If there is an exposure and I saw cases or a certain situation or such, I think about it, and sometimes it doesn’t have to be me who’s in the situation but someone else. I think about it and what they are thinking about. But not always because I would be overthinking.’* [PF3].

Some students took their reflections out of the college context to other real-life situations, as in the following examples:

*‘It has happened to me in real life. I related what I studied with a situation—I mean the clinical relation of the lecture. It was kind of a fracture, and my diagnosis was right. We had the lecture the day before the incident or a day earlier.’* [P3M].

In contrast, some students focused their reflections on their study issues and mainly on the solutions to those difficulties, for example, the following:

*‘For me, any problem facing me related to time management or difficulties or any studying issue, I preferred to talk with seniors because they have been through the exact same experience as mine, at the same college, and they have been taught by the same doctors as me, and this benefits me 200%.’* [P2F].

*‘If I find it difficult to concentrate, I will look for solutions such as reading from a book, reading from a website, and watching videos.’* [P1F].

*‘Block arrangements and the correlation of the information between blocks.’* [P5M].

*‘I remember that case very well, because we linked what we studied with what we saw.’* [P3M].

*‘We had a field visit. They have connected my knowledge with reality, especially with topics related to contamination and vaccines. It was years ago, but I remember it because it cleared up the picture.’* [P4M].

*‘The CDs which in general have helped me and made me think especially in differential diagnosis. I wish it is in all years, not just from fifth year.’* [P1F].

*‘We don’t have a university hospital, so the university should do something alternative to have clinical exposure and see patients in the earlier years.’* [P1F].

*‘It was patient history and clinical examination. Yes, I learned about them in basic science, but when I practiced them, they were different. I think if the university took advantage of the students, instead of us studying how to take a history and do a clinical examination theoretically, they could the students go to summer training, and we would benefit 100%.’* [P1F].

#### Extracurricular activities

The students described an issue of poor awareness of extracurricular activities early in their learning journeys. A lack of these activities and support was also mentioned as an issue, as described in the following:

*‘As seniors, we didn’t know about them, and it is a requirement to participate in a student club for our CV. We didn’t know till this year.’* [P5M].

*‘So, I feel we lack this in our college, and we focus on studying. We don’t have student activities to motivate us and entertain us. Everything is under academic pressure, and the students will burn out, and they will not have the ability or passion to participate or represent the college of medicine in other forums. It is such a waste of excellent potential.’* [P3F].

*‘I wanted to establish a student club similar to the KSU [King Saud University] student club…. I didn’t get the university support and had a financial issue.’* [P3F].

#### Academic atmosphere and resources

In regards to facilities, there were some issues raised contrasting the old and new building, some were related to the separation of the male and female student campuses, and others related to the management of facilities:

*In the old building, we had a public library. The atmosphere was actually encouraging you to study during your free time. Now we understand that the building is new, and this is the third year since we moved in. But, we hope that the future is better regarding this point.* [P4M]

*We were moving constantly between the old college building, theater building, and the new college building. This was in regard to labs. We didn’t get the full advantage of them, and the lack of labs, especially for female students, and we were unsettled in one place. We have moved constantly.* [P3F]

*We don’t have a garden in our building. We can’t sit outdoor. We’re always indoors from 8:00 AM to 2:00–3:00 PM, and we are forbidden to go out and do anything. We don’t have a place where we can breathe fresh air.* [P4F]

*‘If I compare it with another library in a different place, there is a difference. We have shortages in our resources, but what I see is, it is enough, and I don’t know. Additionally, nowadays, everybody is headed for software and soft copies. Honestly, now is different than three years ago; everyone has an iPad, which contains uncountable resources.’*

The clinical posting in the local hospital had an impact mainly on the female students, with comments regarding the perceptions of inequality in the opportunities to learn due to gender:

*‘I mean in terms of cases in King Khalid Hospital are so limited, and some of the doctors were not cooperative with us. The training at King Khalid Hospital was not the best. But there wasn’t inequality regarding our gender.’* [P1F].

As medical students, their focus was more on the clinical teaching-related factors that hindered their learning, such as limited clinical exposure. For most of their issues, they tried to link them to the setting (where they had limited clinical exposure in a small city hospital), gender difficulties due to cultural barriers, time management, and the number of students:

*‘In terms of clinical exposure, we suffer. Some modules, for instance, are also practical, but we see the patient just once or twice during the module. This is due to several reasons, such as the hospital here, patients are few in number, and we don’t have a university hospital’* [P1M].

*‘Sometimes, it is a time matter and wondering how to study clinical skills in the college, then go to the hospital to see what we studied.’* [P4M].

*‘When I asked to go to a different hospital… unfortunately, in the female department, it was so hard to the extent that I went to the male department to transfer. I really wish it gets easier in the coming years.’* [P1F].

In contrast, some students acknowledged the effort that the college was making to overcome the limited clinical experience and appreciated the environment and resources, but with some critiques regarding certain facilities. This was shown in the maturity of the approach of some senior students as they advanced in their learning:

*‘Clinical exposure… this matter is not only the students’ responsibility but also the responsibility of the college and the students at the same time. The college is trying to expose us and allow the students to do the examination once, twice, or more than that. However, some students are not interested. On the other hand, there are interested students, but the opportunity is not available for them.’* [P5M].

*‘The place, in my view… it is excellent in terms of theater and practicing places, except the library. It has limited resources.’* [P5M].

One issue appraised by most of the students was the safe environment and friendly atmosphere. The students, faculty, and administrative staff exhibit great cooperation, but that can be strained as the number of students increases:

*‘The thing that we are doing is having a friendly meeting with doctors, and that depends on people’s free time. This will assist education more, and the general atmosphere would be more comfortable.’* [P4M].

*‘If the number of students is large and they don’t interact with each other, they won’t know each other. So, if there is group division, after a period of time, the group division changes.’* [P5M].

*‘There is huge cooperation between us and the doctors, and there is assistance and explanation when we ask for it, and they give their time off for us more than it is required from them, which is great.’* [P2F].

*‘The environment in general is good, to an extent. Some of the things were better than what we expected…. There is cooperation from the administration generally. For example, simple issues we faced, they have solved for us quickly.’* [P2F].

### Faculty-related factors

#### Role as resource person

The presence of a faculty member has a direct impact on the students: as a role model, a source of applied knowledge and skills that would be hard for the students themselves to perceive abstractly, and an accessible person in the institute with whom the students can easily interact. These various aspects are shown in the following examples:

*‘Some doctors… the knowledge that they gave it to us… I cannot find it in any book or in any video, and the time I spend with them is precious.’* [P3F].

*‘The doctors are cooperative with us. We had good references, and everything was clear as a result. The outcome was very good, but in contrast, the other block was not.’* [P1F].

*‘They really provided a good explanation, especially for the clinical examination. It is impossible to forget how some lecturers explained clinical examination to us in a great and excellent way.’* [P1F].

*‘For example, during the breaks, I might find a certain doctor, and I’m free for half an hour. I sit with him and share ideas, or he explains something for me that will strengthen our relationship.’* [P4M].

*‘For clinical exposure, the faculty members have played their role and tried. There is a lot of bedside teaching in all the modules, and they have offered us a lot.’* [P4M].

Although the flip side of the potential positive impact of these various contributions is that some individual faculty members may not have the skills to guide the students or are failing to do so:

*‘The problem is in some subjects the doctors are not fully knowledgeable or it’s not their subject. So, the doctor has difficulty in delivering the information to the students and that will cause a decrease of student knowledge.’* [P3F].

*‘I must present in a perfect way. On the other hand, the doctors who are teaching and giving us information are not presenting to us in a perfect way. They read from the iPad. They slide, open the book, and read from it*.’ [P1F].

*‘The office hours of the faculty are a very important point that I want to mention. During the Directed Self Learning DSL, you weather have a meeting with doctors during their office hours, or you search about the topic. We have done it before, and we had time out of the lectures and office hours frame where we went to the doctor, so he clarified some points and explained them to us.’* [P1M].

*‘There is someone called a year coordinator, and he can play a major role, but for us as students, we didn’t feel that.’* [P4M].

*‘Some faculty members, if they knew that you are not interested in their subject (specialty), they won’t answer your questions.’* [P3F].

#### Role as evaluator (feedback provider)

The students appreciated the role of the feedback provided by the faculty on their performance and the impact of this practice on their development. However, they did address the feedback they received and the challenges in seeking feedback and how it was provided by the faculty:

*‘Some of them, they give you feedback privately and explain weaknesses are strengths and what I need to change and to pay attention to some points that will come at residency.’* [P3F].

*‘When I present in their seminar and at the end, they give me feedback, so I develop myself. In reference to their feedback, I see an improvement in the next seminar, but other doctors, they don’t give feedback to their students, and that will lead to lack of improvement.’* [P4M].

*‘If we had better communication, it would be fantastic, and I think it is students’ responsibility…and if you know your seminar’s doctor, you search for him and show him your work.…and give you feedback. He will tell you what you need to add and what to remove.’* [P2M].

#### Role as mentor

Having a mentor is highly appreciated by students, and they admire the effort of the faculty to make it happen, both formally and informally. There is still the challenge of keeping it effective and appropriate for each level of student:

*‘Having mentors is so great, but it needs more commitment because this situation is new to the students…. Honestly, the faculty were committed, and they tried to do so many things, such as, if you want to know your grade, go to your mentor, but I feel we need more sessions with our mentors*.’ [P4M].

*‘Started the semester correctly, regular meetings with our mentors… then it decreased gradually, and it became a routine, just filling out papers or signing papers. Mentors are fundamental.’* [P1M].

*‘Before the pandemic, we organized with two doctors to meet in a coffee shop in the evening, and it was an informal meeting. It was so beneficial in terms of life experiences.’* [P1M].

*‘Also, the mentor has a role in impacting our experience. Doesn’t he? [two students verbally agreed with this after it was said.’* [P1M].

*‘We didn’t have a guide to show us what to do. There are so many things we were missing in the basic years.’* [P1F].

### Social and personal factors

#### Educational challenges

The students illustrated that their ability to cope with stressors of having a variety of educational activities that needed good preparation and often more time was challenging. Some students related those stressors to external factors affecting them, such as the short time frame of the courses, number of classes, nature of activities, and demands of the learning materials.

*‘The experience so far is good from some students’ point of view. One of the problems that we face is the number of classes. Some days, we have five or six classes in one day that will reduce students’ understanding, and there’s no equal distribution for the lectures during that is one of the major problems we face*.’ [P1M].

*‘There is no proportioning with the time. We have, for example, some modules; we will only have eight weeks, and the curriculum needs more time than that. Even in the exam, the quantity of the information is not proportional to the time, and I have been struggling with this issue myself.’* [P1M].

*‘The time of Problem Based Learning (PBLs) and CDs and these sorts of things. Honestly, they are putting us under psychological pressure during this time*.’ [P2F].

#### Students’ interactions

The students’ interactions with each other have an important impact (positively and negatively) on the learning environments, as stated in the following examples:

*‘For me, the thing that impacted me was the students. There is motivation and assistance between us. In addition, the faculty did a lot*.’ [P3M].

*‘We notice that there is always a group of studious students, a group of average students, and so on. We need to promote the culture of strong people help weak people. Some students who are advanced academically shouldn’t be enclosed with each other and neglecting other students.’* [P5M].

*‘This phenomenon causes problems between students. For example, one group has a clarification of something or a particular source, and they don’t share it with other groups*.’ [P1M].

*‘Comparing us with the female side, they always tell us that female students got higher marks then you and your level is less than female students’ level, and we struggle with this.’* [P1M].

*‘The second point that I feel helps is the relationships between the students. If the batch is connected and gathered, and they are helping each other, it is different than if they are doing silly actions or hiding information from each other. This will affect them. If there is group work and the students are helping each other constantly, it is different than if they are fighting each other.’* [P5M].

#### Psychological and financial support

Having a supportive context in the matter of psychological or financial support was also addressed as an issue impacting the learning environment:

*‘Secondly, we lack psychological consultations and a clinic in the university, or at least, they could provide consultations and access to them*.’ [P3F].

*‘Not just a program discount, but in general, some universities provide this to their students for free. They contract with shops, for example. Shops provide medical tools or scrubs*.’ [P1F].

## Discussion

This study was conducted to explore the teaching and learning environment at the College of Medicine at Majmaah University. Insights from focus groups shed light on students’ experiences and perspectives within the educational context, revealing key themes in education, faculty interactions, and social as well as personal factors.

### Educational factors

In the context of education, the importance of relevance in teaching methods cannot be overemphasized. Students in the current study valued teaching methods that effectively bridged theoretical concepts with practical application, enhancing the relevance of their learning. This finding was consistent with those from other contexts in which studies examined the dichotomy between perceived educational value and task engagement, as reported by various researchers [33–36]. Learners perceiving a separation between learning and practical work tend to engage selectively with tasks, influenced by their perceived educational value.

This aspect from educators’ was found in earlier literature where educators were required to make frequent decisions regarding what information to incorporate based on its relevance to the curriculum and school policies [37]. This prescriptive nature of relevance aligns with the desire of students for learning that resonates with reality.

In addition, the findings revealed that students want an active role in decision-making regarding their learning, which hints at anticipated relevance, as mentioned by Diekema and Olsen [37]. Thus, the relevance of content and the perceived educational value of tasks are central to the learner’s experience. While the current study focused on the students’ perspectives, the literature suggests that both educators and students have unique insights to offer in this respect, and the bridge between theory and practice can only be truly constructed when these insights are aligned.

The results of our study highlight the diverse ways in which students engage in self-reflection. Some contextualized their reflections within the specific phase of their medical training or the extent of their clinical exposure. Others extrapolated their learning to real-life scenarios, which is relevant to the previous point of perceiving the value of learning, while another group focused exclusively on academic difficulties and their potential solutions.

Schei, Fuks and Boudreau [34] illustrate this concept of reflection well, noting its potential in addressing medical education complexities, though acknowledging challenges posed by human cognition. Schei and colleagues argued for a form of reflection in medical education that results in wise practice, encompassing both self-awareness and self-correcting behaviors, which aligns with our findings in which the students reflected on solutions to their academic challenges.

Hargreaves [38] offers an interesting perspective, suggesting that reflective competence can be developed progressively throughout an undergraduate program. This concept provides a plausible explanation for the variations observed in student reflections. As students move through different stages of their education, the nature and depth of their reflections evolve.

Griggs [39] explored the challenges associated with assessing reflective practices in medical education. Our study’s findings resonate with this, as during the focus group, the reflections leaned toward discussing personal experiences. Griggs’ emphasis on storytelling as a catalyst for deeper reflection also aligns with our observations. When students relate their learning to real-life situations, they are, in essence, crafting a narrative that might foster more profound reflection.

Students reported a noticeable lack of information and encouragement regarding participation in extracurricular activities, which the literature has indicated as important in fostering various essential practical and analytical skills and attitudes, such as leadership, commitment, responsibility [40], problem-solving, and essay-writing abilities [41].

Extracurricular activities has been reported foster student-community interactions, thereby enhancing learning experiences [40]. However, in the current study, the students felt that there was inadequate support for extracurricular activities, both financial and organizational, from the university, echoing the barriers identified by Fuji [40], such as a lack of support, limited positions, and poor availability of physical and financial resources. Students’ feelings of burnout and academic pressure find echoes in existing literature on the subject. which mentions poor time management and the risk of lowered academic performance as potential barriers to participation in extracurricular activities [42].

The findings from the current study and the literature collectively advocate for a more supportive environment for extracurricular activities in medical education.

The results illustrate various issues and positive aspects related to the educational facilities and environment experienced by students. A noticeable concern is the inadequacy of certain facilities amenities, such as a public library and outdoor spaces. Female students, in particular, highlighted issues like insufficient lab facilities and a perception of unequal distribution of resources during clinical postings at local hospitals. Similar results were identified by other studies that emphasized the importance of well-equipped educational environments with necessary facilities and resources for students’ motivation, participation, and self-regulation in learning processes [43, 44].

However, a new educational setting has also been credited for fostering a safe and cooperative environment where students, faculty, and administrative staff effectively collaborate, enhancing the educational experience. The adaptability to technological advancements is evident, with a shift toward digital resources like software and iPads, which seem to partly mitigate the limitations of physical resources. This resonates with the literature’s emphasis on the necessity of up-to-date resources for effective learning [44].

It is thus clear that while there are areas for improvement, particularly concerning facility provisions and gender-related challenges, various elements of the new environment were conducive to fostering a positive and adaptive learning experience.

The students expressed their concerns with the limited clinical exposure and constraints faced due to limited patient availability and the absence of a university hospital. This resonates with the concerns documented in the existing literature. The literature has shown that students exhibit a strong inclination toward hands-on clinical practice [45]. In the current study, gender difficulties, coupled with cultural barriers, further complicated this scenario.

Notably, the educational institution’s role, including that of faculties, has been addressed in literature. For example, there is an emphasis on the indispensability of authentic learning experiences [46]. The students’ reflections highlighted an appreciation for the college’s concerted efforts to improve the deficits in clinical exposure, underlining the institutional responsibility of enhancing the students’ practical learning experience.

Furthermore, the students’ aspirations for enriched clinical encounters were similarly found in Saigal, Takemura [47], emphasizing the influential role of preclinical and clinical experiences in shaping medical students’ specialty preferences and overarching professional paths.

### Faculty-related factors

The multifaceted roles that faculty members play—as resource persons, evaluators, and mentors—are instrumental in molding students’ academic experience was evident in the study results.

Ingraham, Davidson and Yonge [48] emphasized this dimension of the faculty–student relationship, where themes of support and care are paramount. A key aspect of this is the knowledge-sharing that happens outside of structured teaching sessions. This was evident in the results from students’ appreciation for informal meetings with faculty members, where they discussed life experiences in a casual setting like a coffee shop. Such interactions emphasize the importance of faculty as a well-spring of real-world experiences, not just academic knowledge.

As reported by Shim and Walczak [49], the efficacy of pedagogical methods often rests on the quality of the feedback provided. The feedback received from faculty helps students identify their strengths, understand their areas of improvement, and map out future learning pathways. Smith [50] indicated that the relationship between faculty members as mentors and students as mentees is significant in shaping perceptions and satisfaction levels in academic settings. For students, having a mentor is more than just an academic advantage; it is an avenue for holistic growth. Students praised the faculty’s efforts in establishing mentorship and guidance through uncertainties, albeit suggesting the need for more consistent interactions. They also raised concerns about the changing dynamics of mentorship, where initial enthusiasm can sometimes wane, and the interaction can devolve into accomplishing routine paperwork.

### Social and personal factors

Naturally, the students expressed that they encountered stressors during their educational journeys. The results revealed that students found managing multiple educational activities within limited timeframes to be particularly challenging. This aligns with the findings of Weber, Skodda [51], who identified organizational factors, such as inadequate information flow and exam-related issues, as significant contributors to students’ stress levels.

The students highlighted specific external factors exacerbating their stress levels, such as the unequal distribution of lectures, short course durations, and an overwhelming volume of information relative to the time until each exam. These findings align with several studies in Saudi Arabia [52, 53] emphasizing heightened stress levels in medical students due to the intensity of the curriculum and academic achievement. Similarly, Hill, Goicochea and Merlo [54] highlighted system-level concerns, such as administrative and assessment-related pressures, corroborating our findings regarding the impact of external and organizational factors on student stress.

As suggested by Hill, Goicochea and Merlo [54], our study highlights the necessity for structural consideration in medical education to create a more balanced and less stressful academic environment, thereby enhancing students’ ability to cope with the demands of their education.

The students highlighted the role of peer interactions in the learning process. Similarly, Akinla, Hagan and Atiomo [55] emphasized the value of near-peer mentoring in fostering professional and personal development. The students’ revealed that these interactions encompassed collaboration, as well as competition and comparison, and were helpful with their stressors and mental health challenges [54, 56]. In particular, the gender-based comparisons and the withholding of information among groups revealed a competitive environment that can exacerbate stress and hinder collective academic progress.

Students emphasized the essential role of psychological and financial support in enhancing the medical students’ learning environment. This finding is consistent with the broader educational literature that emphasizes the crucial role of financial and mental health services in supporting students’ well-being and academic success [53, 56].

The literature and student feedback collectively emphasize the importance of enhancing the supportive infrastructure within educational environments. Such improvements, including mental health services and financial assistance, are essential for creating a more supportive and conducive learning environment [52].

## Conclusion

This study has highlighted the critical interaction of educational content, faculty involvement, and social and personal elements in shaping students’ educational experiences. A key finding is the students’ strong appreciation for educational relevance and their autonomy in decision-making. Faculty roles, encompassing resources, evaluators, and mentors, are central to shaping both the academic experience and supporting students’ development. Additionally, the necessity to enhance physical learning environments and facilities to meet evolving educational needs is underscored. This study contributes to existing literature by pinpointing specific improvement areas, emphasizing the importance of student involvement and faculty roles in the educational process.

## Recommendations

Based on our study’s findings, we recommend several enhancements to the educational experience in medical colleges. Emphasizing practical applications in the curriculum is crucial to bridge the gap between theory and practice, making learning more relevant and engaging for students.

Student involvement in decision-making processes is essential for fostering a sense of ownership in their educational journey. Implementing structured reflection activities tailored to different curriculum stages can deepen students’ engagement and understanding.

Incorporating practical case studies and scenarios will enable students to relate their learning to real-life situations, enhancing practical knowledge application. Equally important is encouraging student participation in extracurricular activities, which aids in developing essential life skills.

Addressing facility inadequacies, such as enhancing library resources and outdoor spaces, is necessary to create a conducive learning environment. Ensuring equal clinical opportunities for all students, irrespective of gender, and addressing cultural barriers in clinical practice, will promote a more inclusive educational setting.

Strengthening mentorship programs is vital for providing ongoing support to students. Adjusting structural elements of the curriculum, like lecture distribution and course durations, can alleviate student stress, contributing to a more balanced academic environment.

Promoting collaboration over competition among students will foster a supportive educational atmosphere. Finally, enhancing psychological and financial support services, including counseling and financial aid, is crucial for students’ overall well-being and success.

These targeted recommendations aim to improve various aspects of medical education, ensuring a higher quality of learning and student satisfaction.

### Electronic supplementary material

Below is the link to the electronic supplementary material.

**Supplementary material 1:** Topics guide

## Data Availability

The qualitative data used in the current study are not publicly available as the related quotes are shared in the results section, however they can be available from the corresponding author on reasonable request.
