# Supplementary material for: Exploring challenges and perceptions in the learning environment: an online qualitative study of medical students
Source: BMC Med Educ. 2024 Feb 14;24:147. doi: 10.1186/s12909-024-05116-8 (PMC10868022; doi:10.1186/s12909-024-05116-8)
Supplement: Supplementary file 1 — Supplementary material 1: Topics guide [file 12909_2024_5116_MOESM1_ESM.docx]

Topics guide

| **Question** | **Factors listed to be asked according to previous study** |
| --- | --- |
| “what do you think about your experience in the college from teaching and learning perspective let's start with your students related issues as a student. what are the things that affect or impact your experience of teaching and learning in the college?” | 1. Student related issues  - Facilitators & hindrances in learning - Role of self – reflection - Decision about importance & relevance  1. Context related issues  - Socio-academic environment - Extracurricular activities. - Relevance and important of the subject - General atmosphere.  1. Faculty related issues  - Role as mentor or evaluated (feedback) - Role as a resource person |
| Do you think you need to be exposed to clinical practise early? And do you think it will impact your teaching and learning experience?” |  |
| how did that reflect on your learning and teaching experience? Now, let's say you went to the hospital and you saw any fracture for example, how does that reflect on your learning? |  |
| what do you think about the variety of the conditions? is it really influence your teaching and learning experience? |  |
| Is there anything that you have noticed that affected you? Specially, senior students ? something makes a difference in you and your learning? |  |
| I noticed that you talked about resources in two sides in a positive side and in a negative side. Can you explain what you mean?” |  |
| what are things that we can do to motivate high achieving students to help low achievers ? |  |
| How can the student have self-reflection? If the student has gaps, then how to fill them? |  |
| How to manage yourself in order to have an excellent experience of teaching and learning in college? |  |
| can you explain more how your peers affect your experience ? |  |
| what is your evaluation of your learning experience at Almajmaah University College of Medicine? if somebody wants to enroll in the college what's your reply to her will be?” |  |
| can you explain more about those three factors, how did they impact your learning experience ? |  |
